# Hypoxia potentiates cytotoxicity of LPS-activated microglial BV2 cells in vitro by synergistic effects on cytokine and nitric oxide secretion

**DOI:** 10.1186/2194-7791-2-S1-A20

**Published:** 2015-07-01

**Authors:** Susan Jung, Daniel Frey, Florian Brackmann, Mandy Richter-Kraus, Regina Trollmann

**Affiliations:** Department of Pediatrics, Neuropediatrics, Friedrich-Alexander University Erlangen-Nuremberg, Germany

## Background

Microglial activation due to a variety of stimuli results in secretion of high levels of neurotoxic substances including pro-inflammatory cytokines, nitric oxide (NO), and reactive oxygen species. Clinical studies indicate a crosslink between inflammatory and hypoxia-regulated pathways suggesting that bacterial infections may sensitize the immature brain to hypoxic injury.

## Methods

BV2 cells were exposed to lipopolysaccharides (LPS, 1 × 10^5^ EU/ml for 24h) and hypoxia (1% O_2_ for 6h). Cytokine and NO release was quantified by ELISA and the Griess reaction, respectively. Cytotoxicity was determined in MTS cell viability assays.

## Results

Activation of BV2 microglial cells by LPS exposure stimulated significant and persistent production of NO, IL-1β, IL-6, and TNF-α. Even after LPS removal, ongoing NO and cytokine secretion could be observed. While hypoxia alone mediated exclusively a significant, short-term increase of IL-1β, oxygen deprivation enhanced LPS-induced secretion of NO, IL-1β, IL-6, and TNF-α significantly. Surprisingly, pre-stimulation of BV2 cells by hypoxia prior LPS exposure abolished microglial activation suppressing LPS-induced NO production. Hereby, cell-free supernatants derived from LPS-activated microglial cells exhibited a stronger cytotoxic effect in glial and neuronal cells than LPS exposition per se (P < 0.001). Again, hypoxia potentiated LPS-induced cytotoxicity.

## Conclusion

Present data prove that i) the outcome of hypoxia is determined by the microglial activation status and that ii) LPS-induced soluble factors rather than LPS are mediators of microglial neurotoxicity under conditions of hypoxia in vitro. Activation of pro-inflammatory pathways may sensitize microglial cells to promote hypoxia-induced injury of the developing brain. Consequently, our findings may promote neuroprotective therapeutic strategies in the field of perinatal brain injury.

